# Integration of IoT Sensors to Determine Life Expectancy of Face Masks

**DOI:** 10.3390/s22239463

**Published:** 2022-12-03

**Authors:** Vilanya Ratnayake Mudiyanselage, Kevin Lee, Alireza Hassani

**Affiliations:** 1 School of Information Technology, Deakin University, Geelong, VIC 3220, Australia; 2 Car Advance, Ivanhoe, VIC 3079, Australia

**Keywords:** internet of things, sensors, smart masks, personal protective equipment, face masks, mask performance, mask efficiency

## Abstract

Personal protective equipment (PPE) is widely used around the world to protect against environmental hazards. With the emergence of the COVID-19 virus, the use of PPE domestically has increased dramatically. People use preventive and protective mechanisms now more than ever, leading to the important question of how protective is the PPE that is being used. Face masks are highly recommended or mandatory during the time of the COVID-19 pandemic due to their protective features against aerosol droplets. However, an issue faced by many users of face masks is that they are entirely manual, with users having to decide for themselves whether their mask is still protective or if they should replace their mask. Due to the difficulty in determining this, people tend to overuse masks beyond their optimal usage. The research presented in this paper is an investigation of the viability of integrating IoT sensors into masks that are capable of collecting data to determine its usage. This paper demonstrates the usage of humidity and temperature sensors for the purpose of determining a mask’s usage status based on changes in these variables when a mask is put on and taken off. An evaluation was made on the usage of the two sensors, with the conclusion that a humidity sensor provides more accurate results. From this, we present a framework that takes into consideration the factors that affect a mask’s performance, such as time, humidity and temperature, to calculate the life expectancy of a mask.

## 1. Introduction

The COVID-19 pandemic brought forth a global change in personal behavior. The continuous rise in infection rates caused concern for a majority of the global population, with the use of protective measures being practiced by a major part of the population. Personal protective equipment (PPE) is used for the protection of the wearer from environmental threats, with examples of PPE being face masks, gloves, eye goggles and shields [1,2,3].

Due to the COVID-19 pandemic, the usage of face masks was recommended or mandated by a majority of governments due to their protective properties against the COVID-19 virus [4]. Despite the recommendations on using face masks, there is an improper awareness of face mask usage protocols, resulting in situations where people overuse disposable face masks or misuse other types of face masks [5]. This exposes people to threats, which is not ideal in a situation where a person should be well protected against risk factors.

The use of smart technology offers potential solutions to these problems. A traditional face mask is a simple passive device that would filter the air before it reaches the wearer’s mouth or nose. With the integration of smart technology, a traditional mask can be converted into an active or ‘smart’ device that would use data from its surroundings to inform the user of the mask status and even perform actions that would enhance the protection a wearer would receive.

This aim of the research presented in this paper is to develop a framework to evaluate the use of sensors in identifying if a mask is still usable as a protective measure. We have approached this by investigating the factors that would affect a face mask and how IoT sensors can be used to determine the usage time of a mask.

The key contributions of this work are as follows: (i) an investigation into the factors affecting the performance of face masks, (ii) the development of a framework for evaluating the viability of sensor-enhanced PPE masks, and (iii) an approach for determining the life expectancy of a face mask based on the factors affecting a mask’s performance.

The remainder of this paper is as follows. Section 2 provides a review of the existing work and identifies gaps in the area. Section 3 describes our approach for using a smart mask for determining the current mask lifetime. Section 4 describes the experiments that were performed to evaluate the viability of this approach. Section 5 presents some conclusions and describes potential future work.

## 2. Literature Review

Personal protective equipment (PPE) can be identified as any item a person wears to keep themselves safe from environmental hazards [1]. This can include face masks and respirators, face shields, gloves, gowns, head covers and shoe covers [3]. Wearing PPE ensures that the user is guarded against any risk to their health and safety. Likewise, the consequence of not using PPE properly can threaten a person’s safety in the environment they are in [2].

Face masks are a type of PPE that are used to protect the wearer and others from respiratory infections and/or other forms of pollutants in the air [6]. Although face masks were not used widely domestically in previous years, with the emergence of COVID-19, there has been a significant increase in the demand and usage of face masks [7]. The inability to maintain social distance in certain environments has lead to the enforcement of face masks during the time of COVID-19 [8]. The recommended usage of a surgical single-use mask was reported to be up to 4 h. It should be disposed of after 4 h or if the mask’s filter becomes moist and wet in order for the wearer to stay protected from a respiratory infection such as COVID-19 [9,10].

### 2.1. Challenges with Traditional Face Masks

During COVID-19, a large proportion of people carried a mask with them when they went outdoors, but generally, single-use face masks did not get disposed of after the recommended time unless the wearer visited a high-risk setting, such as in a hospital [5]. This is not ideal in a high-risk situation such as with the COVID-19 pandemic and shows the lack of awareness on mask usage. The cost and inconvenience of purchasing disposable face masks has also added to the issue of people reusing their single-use face masks [8].

### 2.2. Factors Affecting the Performance of Face Masks

The effectiveness of face masks depends on various factors that can be considered to be the determinants of the performance of face masks. Mask performance can be categorized into the efficiency of the filtration of a mask and the breathability of a mask [11]. The filtration efficiency defines how well the filter keeps internal and external aerosol droplets from passing through to the mask wearer [12]. The breathability of a mask is a measure of how resistant the mask is to inhalation and exhalation. The more breathable a mask is, the easier it is for the wearer to inhale and exhale safely without ingesting too much carbon dioxide left on the inside of the mask [13].

The filtration efficiency of face masks and the factors affecting it is the most important factor in determining the current mask usefulness. There are several factors that can affect the filtration efficiency of a face mask, including the mask type, particle size, wearing time and respiratory volume. The mask type refers to the material the mask is made with, if it is disposable or reusable and the material of the filter. Based on how well the material filters out aerosols and droplets, the type of mask being worn will determine the performance of the mask in protecting the wearer. The fit of the mask is an important determinant of the performance as a mask being too tight or loose can result in gaps in the mask seal.

Single-use masks are recommended to be safe for up to 4 h of wear, after which the mask may not effectively protect the user [10]. Experiments conducted on different types of masks suggest that cloth masks may have a safe wearing period of up to 2 h, with single-use masks being protective for up to 8 h without significant reductions in the filtration efficiency [12].

Despite the time and mask type being the most significant factors in mask performance, there are certain factors that impact the efficiency of the filtration to some extent over time, which are as follows.

- Moisture—If a mask filter is too moist, it can no longer effectively protect the user. Over time, the mask can get overly moist if the wearer coughs or sneezes while the mask is worn.
- Humidity—Breathing out increases the humidity level (as seen during the first experiment). This can have an effect on the wetness of the mask’s filter (moisture). The respiratory volume or rate can affect how differently the mask deteriorates for different people.
- Air pressure—A well-fit mask will have air pressure between the inside of the mask and the face. If the fit of the mask changes (stretches or tears) during the period of wearing it, then the air could bypass around the mask. Coughing and sneezing can also affect the fit of the mask temporarily, giving an opportunity for threats to pass through the mask.
- Weather conditions—Wearing a mask for long periods of time when it is raining increases the chances of the mask getting wet, thus reducing its potential lifetime.

### 2.3. Integration of PPE and IoT

The IoT, or Internet of Things, is used to refer to a network of ‘things’ that use sensors and software to connect with other devices and systems to exchange data and resources [14].

Smart PPE is an emerging market, with manufacturers applying smart technology to develop PPE using technology such as the IoT and Artificial Intelligence (AI) [15]. COVID-19 has driven healthcare and front-line workers to use smart devices and wearables to detect their own health and the health of potentially infectious people.

#### 2.3.1. Smart Masks

The purpose of a smart mask is to protect the user from external threats and risks, similar to that of a traditional face mask, and there will be some form of IoT or technological integration involved [9,15]. Smart masks can also be seen to identify the prodromes of COVID-19, sanitize the mask while charging the batteries combined with the use of several sensors for different functions, such as red detecting coughing, the temperature, humidity and hemoglobin saturation [16]. There have been developments of smart masks that contain a changeable filter with an embedded module to make the masks more reusable [17]. There are also small components that would clip onto a traditional face mask using magnets, turning them into smart masks [18].

#### 2.3.2. Gap Analysis

To evaluate the available and proposed smart masks, a set of criteria related to smart masks will be used. These will determine if a smart mask used a particular approach when designing their smart masks. The following questions were used when evaluating smart masks.

- Is the mask reusable? Does the device consider ideal mask usage times [9,10]?
- Does the device consider how tightly fit a mask is? Is comfort considered [9,10]?
- Does the device have a reasonable battery life? Is the battery light [19]?
- Is the system location/context aware [20]?
- Does the device alert the user when the mask’s filter gets too moist/humid [9,10]?

Table 1 identifies the factors affecting a mask and determining the remaining lifetime of a mask, the re-usability, time factors and filter evaluations.

Table 1 illustrates work in the area of smart masks and the evaluation of their work in terms of the criteria identified. For the purpose of this research, only certain criteria are considered. Most of the material considered the re-usability of the mask/device and the filtration. However, none of the material provides a solution to inform the user of the available lifetime of a mask at any given time. That is, alerting the user about the current status of the masks’ protection is not being considered. The ideal mask usage times and whether a user is wearing the mask is of high significance due to the fact that masks lose their ability to protect the wearer after a certain time period, or depending on how worn out or affected the filter on the mask is [9,10]. These factors have to be considered in order to ensure the protection of the user. It is imperative that masks are worn by users for as long as the mask remains protective. The gap in the research has been identified as a solution that considers the factors affecting a mask’s protectiveness in order to assess a mask’s current lifetime using the IoT.

## 3. An Approach to Using Sensors to Determine PPE Mask Lifetime

This section proposes an approach for investigating the feasibility of developing a modular device that would retrofit onto traditional PPE face masks, providing a data collection functionality. The results from the data collection can be analyzed and integrated into an equation for calculating the lifetime of a mask.

Traditional face masks have limitations in terms of the protection they can provide to a user. Based on general mask usage patterns, there is widespread usage of disposable masks, as opposed to reusable masks, but in the same fashion as a reusable mask. Therefore, it is imperative that people are aware of how safe their disposable mask is during its usage. This research will focus on building a framework that can be used when building solutions that provide users with the necessary information about the current efficiency of their mask and the estimated lifetime of the mask. The following important steps were identified and will be used to inform a potential solution to this problem.

### 3.1. Using a Sensor-Enhanced Mask to Collect Environmental and User Data about PPE Mask Usage

A sensor-enhanced PPE mask can be used to collect different types of data related to its usage. The collection of data can be performed in different environmental or physical settings for the mask wearer and the data can be read from sensors in order to perform calculations and an analysis on the collected data. If a user is currently wearing a mask or taking it off, the change in this status can be derived with the changes in the collected data from the mask by processing and analyzing it. The output of this is to have datasets relating to mask usage.

Building a sensor-enhanced mask requires the right sensors that are able to fit on a face mask, which is essentially a small piece of electronics that should fit on the wearer’s face. Small, light-weight sensors that collect and log data related to the changes that occur within a mask are suitable for the process of data collection in this regard [26]. Data that are read from sensors directly relate to the current context that the sensor is in. Having sensors mounted on a mask provide the ability to collect interesting data patterns and make connections between the user’s actions and the changes in the data variables. When a mask is worn, with breathing patterns, the temperature inside the mask varies with the ambient temperature outside, making it a good measure of the mask’s status [27]. The humidity variable was identified as a measure of mask usage due to the change in humidity when a mask is worn or taken off [26].

### 3.2. Using Sensor Data from Multiple Sensors to Understand Mask Usage

The data collected from a sensor-enhanced mask can be used to make calculations that use patterns to determine if a user is currently performing a particular action. This can be especially useful when detecting if the mask is being worn or taken off. By using different combinations of datasets, the most efficient method of detecting the mask in-use status can be derived using the concept of response time. The result is a sensor-enhanced mask that can also detect whether a mask is being worn or not with the least latency in the response time.

The data that are collected from the mask can first be used independently. Because temperature and humidity data are being collected, the temperature and humidity will be used on their own to check if a mask being worn or taken off can be detected with a reasonable response time. Using the results as comparative figures, both temperature and humidity can be used in combination to see if the resulting mask detection algorithm provides a better response time than when the variables are used on their own. This will result in a sensor-enhanced mask that can also detect whether a user is wearing a mask or not with the least amount of error time.

### 3.3. Deriving the Current Lifetime of a PPE Mask from Sensor Data

Using the several factors that affect the efficiency of a mask, we propose an approach to determine the amount of time remaining for a mask before it has to be replaced or sanitized. This is based on how each factor affects the current status of the mask. The effect that these coefficients can have on a mask’s efficiency and lifetime is analyzed and used to develop the framework. From this approach, software can be created that considers the remaining lifetime of a mask and informs users about their mask.

The first step to be taken for this is to identify the factors that can affect a mask’s protectiveness over time. These can be used in an equation that considers the weighting of each factor and reduces the total lifetime of the mask according to these coefficients. This will result in a formula that can be used to identify the current lifetime available for a mask using the variable coefficients.

### 3.4. Proposed Concept

**Figure 1:**
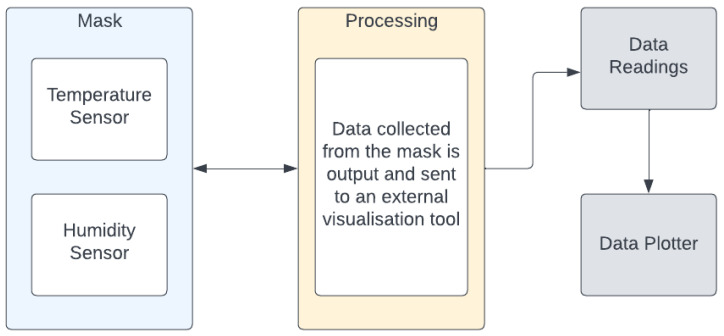
Blueprint Architecture Diagram.

The developed prototype platform is essentially a disposable face mask retrofitted with a temperature sensor and a humidity sensor connected to a microprocessor. Data are collected from the sensors and processed in order to identify patterns in the data. The data can be visualized to manually identify patterns as well. Figure 1 illustrates the architecture diagram for the prototype.

**Figure 2:**
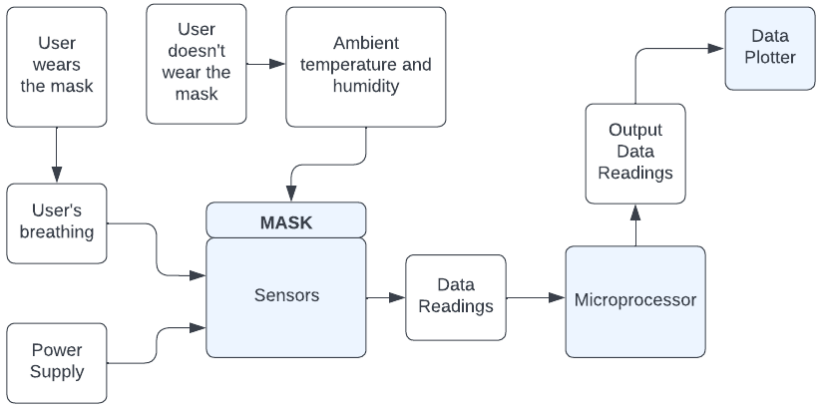
System Diagram.

The sensors on the mask continuously read data from the environment. When the user wears the mask, their breathing leads to changes in the air inside the mask. This, in turn, leads to changes to the temperature and humidity within the mask. The data are read and transmitted to the microprocessor and then plotted on the data plotter. This can be seen in Figure 2, which shows the system diagram for the prototype framework.

**Figure 3:**
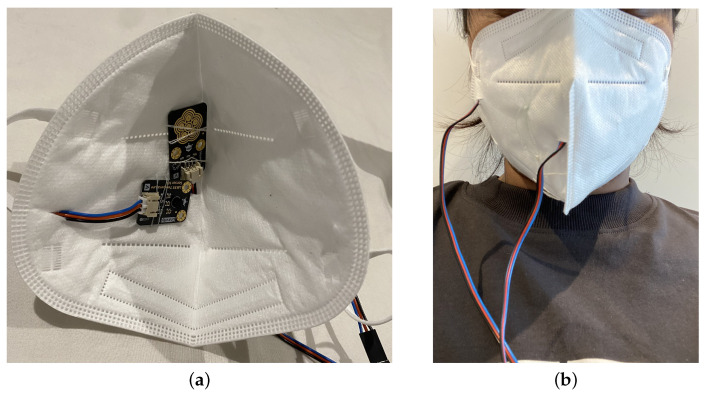
The prototype smart mask: (**a**) inside of the prototype; (**b**) a user with the mask on for the experiment.

To evaluate the approach, a prototype was built as shown in Figure 3. The inside of an N95 face mask is fitted with a temperature sensor and a humidity sensor. Their wires are passed through small slits on the mask. In order to avoid movement of the sensors, the sensors are sewn in place. The sensors are positioned in the spacious area in the middle of the mask. This positioning was selected so that the sensors are not obstructed by the nose or mouth of the wearer. The mask is to be worn in the same fashion as a regular face mask, over the face, covering the nose and mouth. The prototype mask uses off-the-shelf and widely available components. The mask is a standard low-cost N95 mask that costs around 3 USD. The electronics used include temperature sensors, humidity and pressure sensors in the inside and outside of the mask, totaling around 20 USD. The sensors are connected to an Arduino Uno which costs around 10 USD. The prototype costs around 30 USD but would be a fraction of the cost if mass produced.

## 4. Experiments and Evaluation

### 4.1. Data Collection

The initial set of experiments described here focus on demonstrating the data collection using temperature and humidity sensors using the sensor-enhanced mask framework. Different variables were used to collect the data from different contexts. For each experiment, the mask is left idle for 1 min, worn for the next 3 min and left idle again for 1 more minute. Each experiment lasts for a total of 5 min. In order to ensure that contextual mask usage is considered and to improve the accuracy of processing the measured data, the experiments were conducted in four combinations of the following variables: during the day, during the night, with physical activity and without physical activity. The physical activity for the purpose of this experiment was jogging for 5 min prior to the experiment being performed.

**Figure 4:**
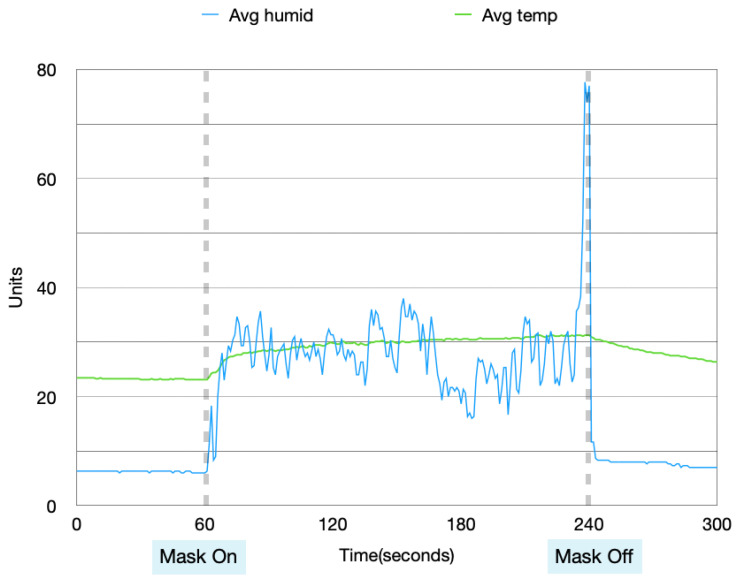
Humidity and temperature collected during the day, with no prior physical activity.

**Figure 5:**
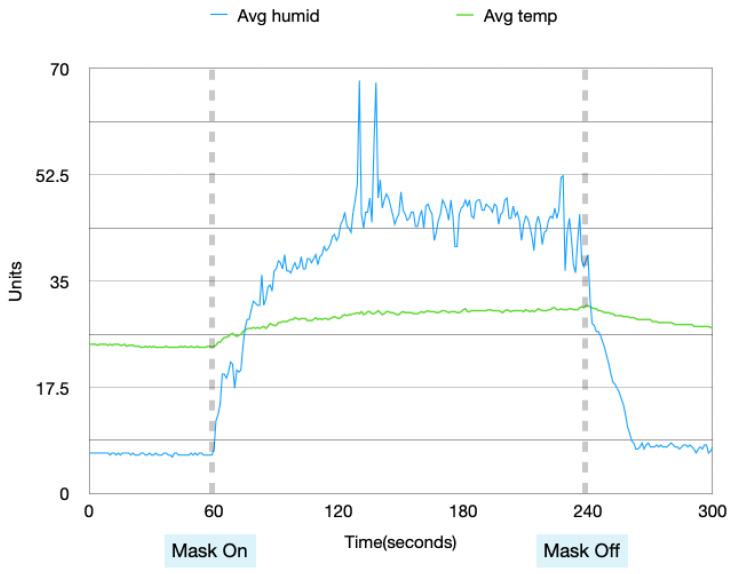
Data collected during the night, with no prior physical activity.

**Figure 6:**
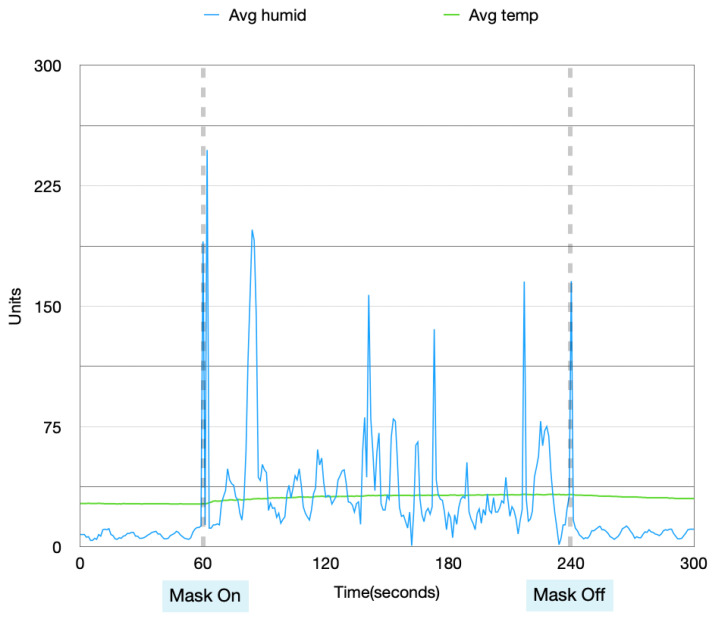
Data collected during the day, with prior physical activity.

**Figure 7:**
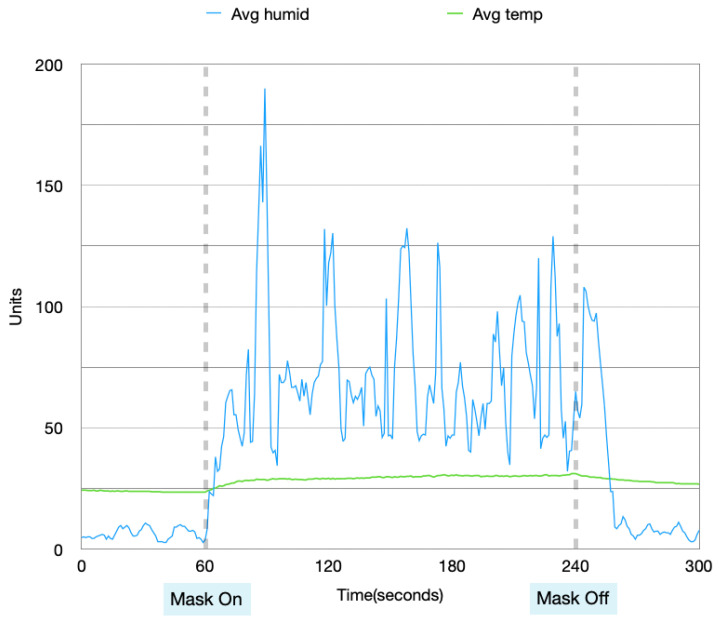
Data collected during the night, with prior physical activity.

Data collected from these four experiments were visualized in order to analyze the patterns in the data when the mask is idle and in use. Figure 4, Figure 5, Figure 6 and Figure 7 show the data collected from the mask in a graphical format.

From Figure 4, Figure 5, Figure 6 and Figure 7, a pattern can be seen in the humidity data. Before the mask is put on, the humidity values are at a low, stable range. Once the mask is put on, the humidity data read large fluctuations and spikes. Once the mask is removed again, the data values return back to stable values. With the temperature data, when the mask is first off, the values are very stable. Once the mask is worn, the temperature gradually increases. After the mask is taken off again, the temperature returns back to a lower temperature very slowly and gradually. These changes in values can be used to find distinctions between when the mask is put on and taken off.

### 4.2. Mask Response Efficiency

This section focuses on the efficiency of detecting if a mask is idle or in use by varying the comparisons made between the data values received from the sensor-enhanced mask. Comparing the data values from the past with values from the present allows significant changes in the values to be detected, which in turn lets the in-use status of the mask be determined. The threshold for identifying if the difference in humidity and temperature of the previous and current data readings reflect a change in mask status was determined based on experimental observation.

The response time (*RT*) is calculated by finding the difference between the timestamp of the device informing the user that the mask status has changed (*RMSC*) and the timestamp of the mask status changing in actuality (*AMSC*).

(1) RT=RMSC−AMSC

**Figure 8:**
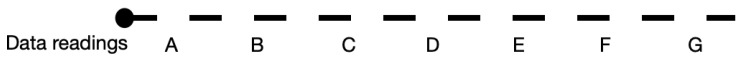
Data Readings Visualized.

The experiments for evaluating the response times were carried out under three categories: 2 s averages, 5 s averages and 10 s averages. These three averages represent the average data being used for each comparison between the previous data reading and the current data reading. Figure 8 shows the results from these experiments. With the assumption that the frequency of the data readings is one per second, for a 2 s average window, the average of values A and B will be compared with the average of values C and D. The same approach was used for the 5 s averages and 10 s averages.

The response time will be calculated using the humidity data and temperature data. Based on the results, the feasibility of a data fusion will also be considered. This can be used to determine the approach that results in the most accurate determination of the mask’s status.

#### 4.2.1. Humidity

The aim of these experiments is to determine the average response time for each humidity experiment and an analysis of this will be performed in Section 4.6.

**Figure 9:**
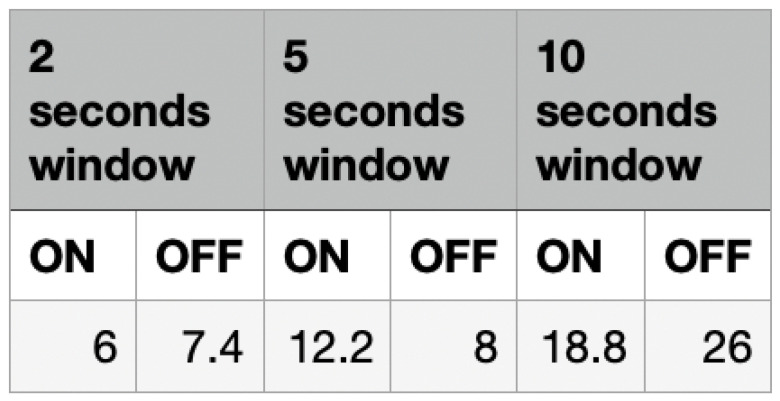
Response Time–Humidity.

Figure 9 shows the response time taken by the mask to determine whether the mask is taken off or put on based on the humidity. The values in the last row of the table show the average experimental results for each of the experiments performed. The 5 s average experiment shows an average response time of 12.2 s for when the mask is put on and 8 s for when the mask is taken off. The 2 s average experiment showed an average response time of 6 s for detecting the mask being worn and 7.4 s for the mask being taken off. The 10 s average experiment resulted in an average response time of 18.8 s for detecting that the mask is worn and 26 s for when the mask is taken off.

#### 4.2.2. Temperature

The average response time for each temperature-related experiment will be detailed in this section and evaluated in Section 4.6.

**Figure 10:**
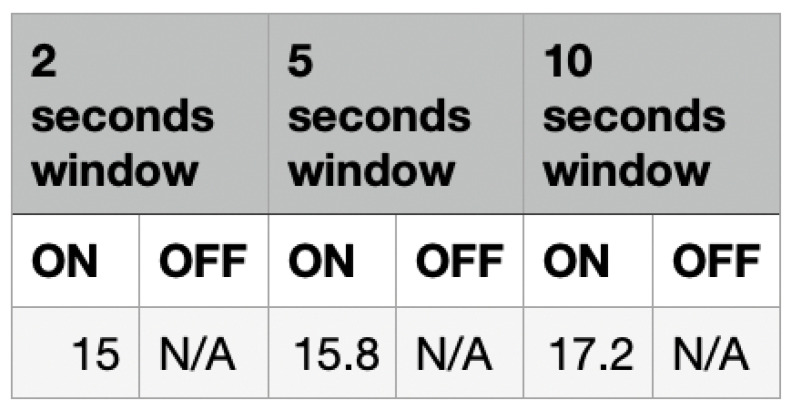
Response Time–Temperature.

Figure 10 shows the average response time taken by the mask to identify the in-use status of the mask. For detecting whether a mask is put on, the 2 s window gave a time of 15 s, the 5 s window gave an average response time of 15.8 s and the 10 s window gave a response time of 17.2 s. The temperature values could not be used to detect whether the mask was taken off due to the slow rate at which the temperature reduces back to its original state (the external temperature).The temperature readings showed a slow change in the data values when the mask was taken off. This slow change produces stable differences between the values, making it difficult to compare the previous and current data values to determine whether a change in the temperature relates to the mask being taken off.

### 4.3. Effect of Breathing on Humidity

**Figure 11:**
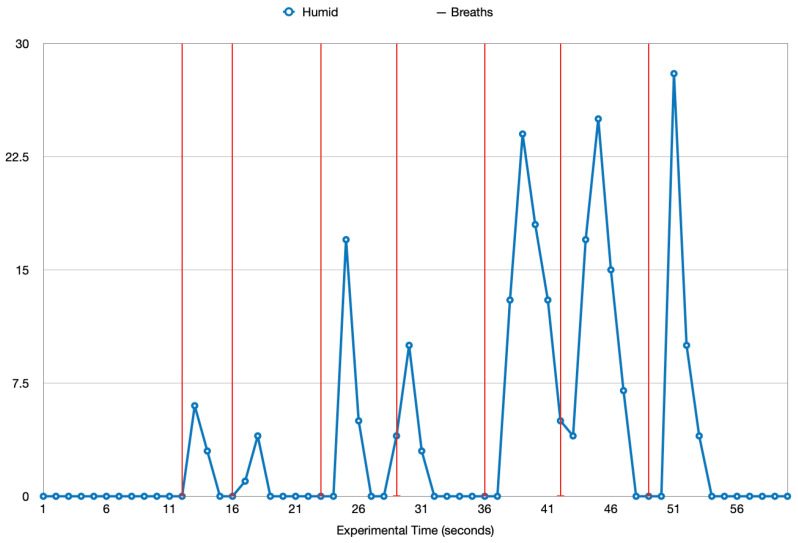
Humidity Readings and Breaths Taken.

**Figure 12:**
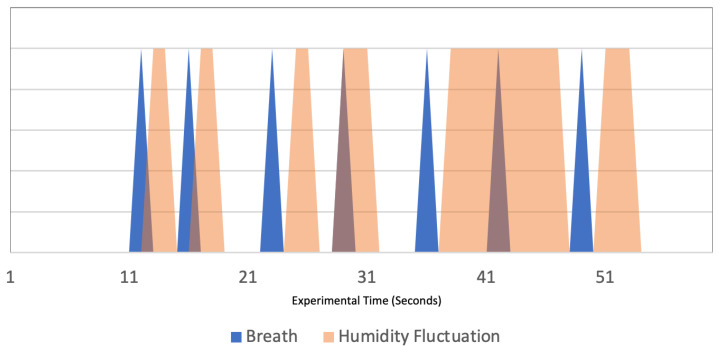
Breathing Patterns and Humidity Fluctuations.

This experiment investigates the effect that breathing has on the humidity inside the mask when a mask is being worn. For the experiment, the mask was worn for 40 s, during which the humidity sensor collected data from within the mask. The breaths taken during the 40 s were recorded and these two sets of data were plotted as in Figure 11. The seven vertical red lines represent seven exhaled breaths during the minute of experimental activity and the blue line represents the humidity during this time. Each humidity data value from the experiment is shown on the humidity line with the circle symbol. Looking at the results, until the first breath was exhaled, the humidity remained at 0. After the first breath, the humidity spiked up. A few seconds after the exhalation and the increase in humidity, there is a downward fluctuation in the humidity. This pattern can be seen after each of the exhaled breaths, highlighting the correlation between breathing and the humidity inside a mask. To further demonstrate this correlation, the breaths and the humidity spike were plotted, as shown in Figure 12. The blue area represents a breath and the orange area represents a change in the humidity. A clear relationship can be seen in the humidity change and the breaths because each breath results in a certain increase in the humidity. This gives adequate reasoning for the correlation between breathing and humidity.

### 4.4. Effect of Usage on the Internal Humidity

The aim of this experiment is to compare the humidity data from a used mask and a new, unused mask to look for differences in the readings. The results will be analyzed to identify the efficacy of using humidity as a derivative for the lifetime of a mask.

An N95 mask that was used for a day and a new N95 mask were used, equipped with the same humidity sensors, in the same environment and context. Two experiments were conducted, one for each of the masks. Each experiment was carried out for 120 s with the masks being idle in the first 10 s. The rest of the 110 s were recorded with the mask worn.

**Figure 13:**
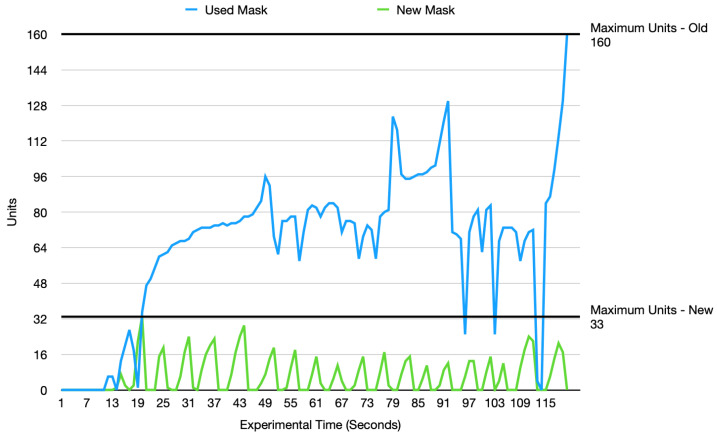
Humidity: Used Mask Vs. New Mask.

Figure 13 shows two lines representing the humidity data readings from a mask that was used for a day and a brand new mask. The used-mask readings are quite high compared to the new-mask readings, which appear to be very uniform fluctuations. A clear distinction can be made on the internal humidity of a used mask and a new mask. This distinction allows for humidity to be used as a strong indicator for the lifetime of a mask among other indicators, such as temperature.

The results from the experiment were plotted as in Figure 13. The blue line represents the humidity readings from the used mask and the green line represents the humidity data from the new mask. The two horizontal black lines at 33 units and 160 units represent the maximum experimental humidity recorded by the new mask and the used mask, respectively. At a glance, the used-mask readings appear to be quite high compared to the new-mask readings, which are all less than or equal to 33 units. The new mask also appears to have uniform fluctuations in contrast to the used mask which recorded more variations in the data values. Using this analysis, a clear distinction can be made between the internal humidity of a used mask and the internal humidity of a new mask. As a result, the internal humidity of a mask can be used as a strong indicator of how old and worn out a mask is.

### 4.5. Analysing Mask Lifetime

From Section 2.2, we can see that there are several factors affecting the mask efficiency and performance. To develop an equation for deriving the mask’s lifetime, the following factors will be used.

1. Wearing Time (T).
2. External Humidity (Ehum).
3. Internal Humidity (Ihum).
4. Temperature (Temp).
5. Air Pressure (Air).

The available lifetime (AL) of a mask can be considered to be at the highest value point before a mask is worn for the first time, with the addition of any factors reducing the available lifetime of a mask.

Each of the five coefficients above are assigned individual weights, namely TW, EhumW, IhumW, TempW and AirW. These weights are variable percentage values that can change depending on the impact of the relative coefficient on the lifetime of the mask. The following equation can be derived from these coefficients.

(2) $$\begin{array}{cc} AL= & \left\{100-((T*TW)+(Ehum*EhumW\right)\\ \; & +(Ihum*IhumW)+(Temp*TempW)\\ \; & +(Air*AirW))\} \end{array}$$

### 4.6. Determining Mask Lifetime from Sensor Data

The aim of this section is to identify patterns in the data being collected from a PPE mask, analyze the data being read from the sensor-enhanced mask and derive the status of the mask in order to allow this data to be combined with other sensor data and evaluating the framework formula identified in Section 4.5.

The data were collected using sensors mounted on a disposable face mask and analyzed to identify patterns. A noticeable occurrence was the rise in temperature and humidity when the mask was put on. For the humidity, the increase was similar to frequent spikes in the values, whereas the temperature data experienced a more gradual increase. When the mask was taken off, both the data values reduced, with the temperature going through a gradual decrease and the humidity going through a more steep decrease. This behavior of the temperature and humidity prove that they are useful variables for determining the mask status, thus providing a basis for identifying the total time of mask usage.

#### 4.6.1. Humidity

**Figure 14:**
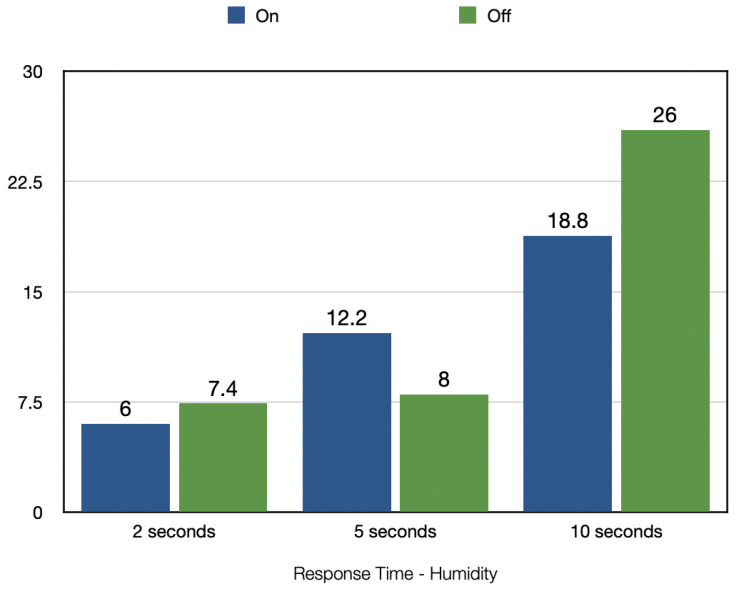
Visualization of Response Time–Humidity.

The average response time taken by the device to detect the status of the mask with the use of a humidity sensor was detailed in Section 4.2.1. Figure 14 visualizes this average response time so that it can be analyzed. The 2 s average readings approach appears to have the least response time in comparison to the other approaches for detecting the ‘On’ and ‘Off’ status. A likely reason for this is the rigid and fluctuating nature of the humidity readings when the mask is put on compared to the stable values when the mask is taken off. This clear distinction allows for easy comparisons between the data values to determine if a mask is in an ‘in-use’ status or not.

#### 4.6.2. Temperature

**Figure 15:**
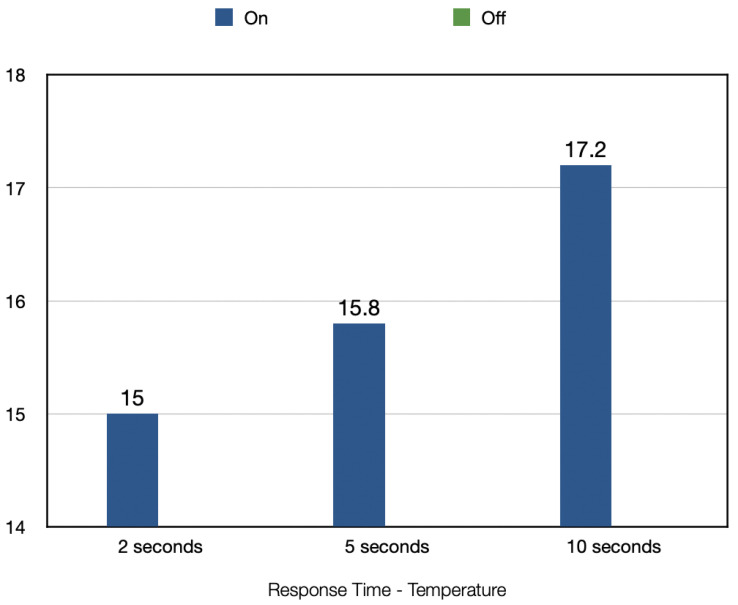
Visualization of Response Time–Temperature.

The aim of this section is to analyze the absolute differences in the temperature data as shown in Figure 15. Looking at the results, for the temperature data, the 2 s window approach had the fastest average response time of 15 s. If only the temperature data are to be used for detecting if a mask was put on, then this would be the best approach. However, the temperature readings cannot be used to detect if a mask is taken off due to the slow and regressive change in the temperature readings. It is difficult to make comparisons between the previous data and current data in a fast and efficient manner due to this slow change in the variable.

#### 4.6.3. Humidity and Temperature

The humidity and temperature data can be combined in order to take advantage of the better functionality of each variable. Comparing the results in Figure 14 and Figure 15, it is clear that the humidity experiments gave the fastest response times for taking the mask off and putting the mask on.

### 4.7. Discussion

The evaluation of the approach proposed in Section 3 was carried out in the form of experiments and investigations. The results from the first set of experiments gave an insight into the use of IoT sensors to collect data from a mask in order to identify the changes in the data readings based on mask usage changes.

The second set of experiments provided an understanding of how the temperature and humidity data being collected from the mask can be used to determine if the mask is currently being worn or not. The humidity experiments showed promising results, with the lowest average response time for the program to detect if the mask is being worn or taken off being 6–7.4 s. The temperature experiments demonstrated slower response times, with the fastest average response time for detecting if the mask is worn being 15 s. Because the temperature readings could not be used for detecting the mask being taken off, a clear decision can be made about the most accurate algorithm for determining the mask’s status based on the results obtained. Using the humidity data alone would be sufficient for determining the mask’s status. These results can now be used for keeping track of the length of time the mask is in use.

The equation for deriving the remaining lifetime of a mask was developed, and a formula was created that considers several factors affecting a mask’s efficiency. The use of weights provides a significance-based approach that helps in determining if a particular factor affects a mask’s efficiency more than others.

## 5. Conclusions

This paper investigated how traditional PPE face masks may not provide the adequate amount of protection to a user depending on how the mask is used. The focus of the paper is on trying to provide technical solutions to solve the solution of PPE overuse, in the case of this paper, with wearing face masks too long. With the use of the IoT, a sensor-enhanced mask was developed that can read humidity and temperature data. The data being read are then processed and the in-use status of the mask is derived using the changes in the data values.

Using this technical approach to retrofitting and collecting face mask use data, a framework for calculating the lifetime of a mask was developed and a general equation was created that takes into consideration the different coefficients that represent factors affecting the efficiency of a mask. The framework produced can be used for future development, such as with the development of a standalone smart mask that alerts users of their mask’s current usability.

The approach taken in this paper has proven that it is feasible to retrofit sensors to PPE face masks to create smart masks as prototypes for commercial smart masks. It has demonstrated the use of multiple sensors to collect sensor data and determine mask use. It has provided a framework for estimating mask use using multiple sensor data sources. It has highlighted that the most valuable sensor type for detecting mask use is a humidity sensor. Future work should build on this by considering power consumption, contextual mask usage and the filter; a smart mask can be developed that informs the user about the lifetime remaining for their mask but also uses less power and considers the environment the user is in.

## Figures and Tables

**Table 1:** Evaluation of Related Work.

Smart Masks			
Title	Device Type	Reusability	Filter
A Smart Mask for Active Defense against Coronaviruses and Other Airborne Pathogens [21]	Mask, belt unit	n/a	Active
Innovative Smart Face Mask to Protect Workers from COVID-19 Infection [16]	Mask	Reusable after sanitization process	Active–Passive
Smart mask—Wearable IoT solution for improved protection and personal health [17]	Mask with changeable filter	Reusable mask shell with replaceable filter	Passive
FaceBit [18]	Modular device	The module can be used in any face mask	Passive
Air Plus Smart Mask [22]	N95 mask with attachable ventilator	Mask is single use only, ventilator is reusable	Active ventilator, passive mask filter
Electrospun polyetherimide electret nonwoven for bi-functional smart face mask [23]	Mask	Able to be used daily	Active
Smart face mask with an integrated heat flux sensor for fast and remote people’s healthcare monitoring [24]	Mask	Reusable because filtration is not the main focus	n/a
Design of a Self-powered Smart Mask for COVID-19 [25]	Mask	Reusable as long as energy can be harvested	Active

## Data Availability

Not applicable.
